# Complete Genome Sequence of Cupriavidus sp. Strain EM10, Isolated from Sewage Sludge

**DOI:** 10.1128/MRA.00714-21

**Published:** 2021-09-16

**Authors:** Sun-Wook Jeong, Yong Jun Choi

**Affiliations:** a School of Environmental Engineering, University of Seoul, Seoul, Republic of Korea; University of Southern California

## Abstract

Cupriavidus species have been known as versatile microorganisms in the field of industrial biotechnology. Cupriavidus sp. strain EM10 was isolated from sewage sludges. Here, we report the complete genome sequence of this bacterium, which contains 6,658,510 bp (GC content, 65.12%) and 6,248 genes.

## ANNOUNCEMENT

Members of the genus Cupriavidus, belonging to the family *Burkholderiaceae*, have received much attention as bioplastic producers (1, 2) and potential bioagents for the bioremediation of heavy metal- and xenotoxic compound-contaminated environments (3). Exploring the genome of Cupriavidus sp. strain EM10 can provide knowledge of the underlying mechanisms for detoxifying toxic pollutants and synthesizing valuable biopolymers.

Here, we report the complete genome sequence of Cupriavidus sp. strain EM10, isolated from sewage sludge from a primary sedimentation tank in Seoul, Republic of Korea (37°33′27″N, 127°03′55″E). To achieve this, 1 g sludge sample was added to lysogeny broth (LB) (1% tryptone, 0.5% yeast extract, 1% NaCl) and cultivated at 37°C with agitation (200 rpm) overnight. Serially diluted cultures were spread onto LB agar medium supplemented with 10 mM NiCl_2_ and incubated at 37°C for 7 days to obtain a pure single colony. Genomic DNA was extracted from 10 ml overnight culture using a Wizard genomic DNA isolation kit (Promega, Madison, WI, USA) according to the manufacturer’s instructions.

Genome sequencing was performed by Chunlab, Inc. (Seoul, Republic of Korea). The DNA library preparation was performed using the PacBio DNA template prep kit 1.0 (Pacific Biosciences, USA). Briefly, 10 μg genomic DNA was sheared and size-selected to 20 kb using g-TUBE devices (Covaris, Inc., Woburn, MA, USA). Blunt-end SMRTbell adapters were then ligated. Subsequently, the prepared library was sequenced on the PacBio RS II platform using PacBio P6-C4 chemistry in 8-well single-molecule real-time (SMRT) v3 cells (4). Default parameters were used for all software unless otherwise specified. There were 63,382 reads after sequencing. The total bases, mean length, and *N*_50_ values of the reads were determined to be 971,301,097 bp, 15,324 bp, and 21,194 bp, respectively. The average quality of the reads was 0.84. *De novo* genome assembly was performed using the Hierarchical Genome Assembly Process (HGAP) v2 protocol (5) in SMRT Analysis v2.3.0 software with the setting “genomeSize = 6,600,000 bp.” The resulting contigs were circularized using Circlator v1.4.0, which includes the removal of overlapping sequences corresponding to the beginning and end of each contig (6). The assembly generated four circular contigs, representing two chromosomes and two plasmids, with sizes of 3,607,341 bp (chromosome 1, 65.5 mol% GC), 2,510,418 bp (chromosome 2, 65.7 mol% GC), 327,127 bp (plasmid 1, 59.3 mol% GC), and 213,624 bp (plasmid 2, 61.21 mol% GC), respectively. The genome sequence was rotated to start at the *dnaA* gene or predicted genes near its center. Gene prediction and annotation were processed using the NCBI Prokaryotic Genome Annotation Pipeline (PGAP) v5.2 with the GeneMarkS2+ annotation system (7). The final genome size of Cupriavidus sp. strain EM10 is 6,658,510 bp, with a GC content of 65.12%. Annotation of this genome revealed a total of 6,248 genes, including 5,273 coding DNA sequences (CDSs), 12 rRNAs, 64 tRNAs, 4 noncoding RNAs (ncRNAs), and 895 pseudogenes. This complete genome sequence provides a genetic basis for elucidating the potential of the newly isolated Cupriavidus strain EM10.

### Data availability.

The genome sequence reported here has been deposited at DDBJ/EMBL/GenBank under accession numbers CP076060.1, CP076061.1, CP076062.1, and CP076063.1. The Sequence Read Archive (SRA) accession numbers are SRX11156676, SRX11156677, and SRX11156678 (PacBio). The BioSample and BioProject numbers are SAMN19357574 and PRJNA733020, respectively.
